# Lifelong Physical Activity Prevents Aging-Associated Insulin Resistance in Human Skeletal Muscle Myotubes via Increased Glucose Transporter Expression

**DOI:** 10.1371/journal.pone.0066628

**Published:** 2013-06-21

**Authors:** Tipwadee Bunprajun, Tora Ida Henriksen, Camilla Scheele, Bente Klarlund Pedersen, Charlotte Jane Green

**Affiliations:** 1 The Centre of Inflammation and Metabolism at Department of Infectious Diseases, Rigshospitalet, The Faculty of Health Sciences, University of Copenhagen, Copenhagen, Denmark; 2 Department of Physiology, Faculty of Science, Mahidol University, Bangkok, Thailand; University of Rome La Sapienza, Italy

## Abstract

Both aging and physical inactivity are associated with increased development of insulin resistance whereas physical activity has been shown to promote increased insulin sensitivity. Here we investigated the effects of physical activity level on aging-associated insulin resistance in myotubes derived from human skeletal muscle satellite cells. Satellite cells were obtained from young (22 yrs) normally active or middle-aged (56.6 yrs) individuals who were either lifelong sedentary or lifelong active. Both middle-aged sedentary and middle-aged active myotubes had increased p21 and myosin heavy chain protein expression. Interestingly MHCIIa was increased only in myotubes from middle-aged active individuals. Middle-aged sedentary cells had intact insulin-stimulated Akt phosphorylation however, the same cell showed ablated insulin-stimulated glucose uptake and GLUT4 translocation to the plasma membrane. On the other hand, middle-aged active cells retained both insulin-stimulated increases in glucose uptake and GLUT4 translocation to the plasma membrane. Middle-aged active cells also had significantly higher mRNA expression of GLUT1 and GLUT4 compared to middle-aged sedentary cells, and significantly higher GLUT4 protein. It is likely that physical activity induces a number of stable adaptations, including increased GLUT4 expression that are retained in cells *ex vivo* and protect, or delay the onset of middle-aged-associated insulin resistance. Additionally, a sedentary lifestyle has an impact on the metabolism of human myotubes during aging and may contribute to aging-associated insulin resistance through impaired GLUT4 localization.

## Introduction

Aging is associated with increased development of skeletal muscle insulin resistance and subsequent development of type 2 diabetes [1], [2], [3] however, the mechanisms of age-related decline in glycemic control remain unclear. Skeletal muscle is the main target of insulin resistance and increased prevalence of insulin resistance and type 2 diabetes has been attributed to the modern lifestyle: a diet high in saturated fat and low physical activity [4]. The aging of muscle has also been shown to involve extrinsic factors such as exercise, diet and a sedentary lifestyle [5]. It has been well documented that physical inactivity increases the risk of type 2 diabetes [6] and other diseases. Moreover, a sedentary lifestyle has been shown to interact with secondary aging: aging associated with disease and environment [7], [8]. Secondary aging is known to reduce life expectancy and therefore a better understanding of the counteracting mechanisms of physical activity is necessary. Aging associated decline in insulin sensitivity occurs in both obese and non-obese populations [9], [10] and therefore is unlikely to be dependent on adiposity alone. Additionally, insulin resistance is related to many other clinical factors of aging including skeletal muscle weakness and sarcopenia. Life-long physical activity, such as seen in elite athletes has shown that 60 year old athletes have the same glucose and insulin levels during an oral glucose tolerance test as 26 year olds [11]. It is therefore unlikely that deterioration in insulin sensitivity is an inevitable consequence of aging and is maintained by regular physical activity. Physical activity has been shown to elicit a range of beneficial metabolic and functional adaptations in aging including synthesis of contractile proteins [12], increased mitochondrial function [13], and increased insulin sensitivity [14].

Skeletal muscle consists of different cell types including quiescent satellite cells that are present on the basal lamina of myofibers [15]. Muscle satellite cells act as stem cells and have been shown to be important for muscle regeneration following injury [16], [17], [18]. We and others have shown that muscle satellite cells when differentiated into myotubes *in vitro* retain a “memory” of their (*in vivo*) phenotype [19], [20], [21], [22], [23], [24]. In the current study we aimed to address the effects of aging on the insulin signaling pathway in isolated satellite cells. We further asked whether a lifelong extremely active lifestyle had the potential to counteract an eventual age-dependent decline in insulin sensitivity. Thus, we isolated satellite cells from young healthy males or middle-aged males who were either lifelong sedentary or extremely active, differentiated them into myotubes and measured the effects of physical activity level and aging on the insulin signaling pathway.

## Materials and Methods

### Materials

F10 nutrient mixture (HAM), Dulbecco’s modified Eagle’s medium (DMEM), fetal bovine serum (FBS), horse serum (HS), penicillin/streptomycin (P/S) and Fungizone antimycotic (FZ) were obtained from Invitrogen (Taastrup, Denmark). Insulin (Actrapid) was from Novo Nordisk (Bagsværd, Denmark). Complete mini protein phosphatase inhibitor tablets were purchased from Boehringer-Roche Diagnostics (Copenhagen, Denmark), and protein protease inhibitor I and II were from Sigma-Aldrich (Brøndby, Denmark). 2-deoxy-D-[3H]-glucose was from Perkin Elmer Life Sciences (Copenhagen, DK). Phospho-Akt/Akt (Ser473), phospho-GSK3α/β (Ser 21/9), anti-Akt anti-α-actinin, anti-β-tubulin and p21 Waf1/Cip1 antibodies were from Cell Signaling Technology (Boston, MA). Anti-Myosin heavy chain (MHC) was from Developmental studies Hybridoma Bank (Iowa, IA), Alexa Fluor 488-conjugated goat anti-rabbit IgG antibodies and Alexa Fluor 647-conjugated wheat germ agglutinin (WGA) were obtained from Molecular Probes, Inc (Naerum, DK). TRIzol was from Invitrogen (Taastrup, DK).

### Subjects

Skeletal muscle biopsies from *vastus lateralis* were obtained from either: 1) young, healthy, recreationally active, 2) middle-aged sedentary (sedentary for >10 years, maximum of 1 hour per week physical activity) or 3) middle-aged active (training volume at least 50 km/week running for past 5 years or 10 marathons completed (2 within the last 2 years)) males (n = 5 per group). All subjects were matched for body mass index (BMI), were non-smokers and had no chronic diseases (see Table 1 for subject characteristics). The experimental protocol was performed in accordance with the Helsinki II Declaration and approved by the Scientific Ethics Committee of the Capital Region of Denmark (H-4-2010-111). All subjects were informed and gave written consent before participation.

**Table 1:** Characteristics of muscle satellite cell donors.

	Young (N = 5)	Middle-aged sedentary (N = 5)	Middle-aged active (N = 5)
Age (years)	22±1.1	57±2.5***	56±2.1***
BMI (kg/m^2^)	22.1±0.8	22.8±0.7	23.8±0.8
Fat Free Mass (kg)	65.9±3.4	59.4±4.2	58.7±1.2
VO_2_ max (l/min)	3.8±0.3	3.4±0.3	3.7±0.2
VO_2_ max (ml/kg/min)	47.4±1.3	43.4±1.4 (¤)	48.5±1.9
Fasting glucose (mmol/L)	5.7±0.2	5.2±0.1	5.1±0.2
OGTT 2 h glucose (mmol/L)	6.2±0.8	5.7±0.6	6.6±0.6

Data are means ± SD. OGTT, oral glucose tolerance test.

*** P = <0.0001 vs. young subjects.

(¤)P = 0.07 vs. middle-aged active subjects.

### Human Muscle Satellite Cell Isolation and Culture

Satellite cells were isolated from *vastus lateralis* muscle biopsies as previously described [20]. Briefly, after removal of fat and connective tissue the muscle biopsy was minced into small pieces and digested in buffer containing 0.05% typsin-EDTA, 1 mg/ml collagenase IV and 10 mg/ml bovine serum albumin (BSA) for 5 min at 37°C. Subsequently, digestion solution containing liberated muscle precursor cells were transferred to cold FBS to inactivate trypsin activity. The solution was centrifuged at 800 g for 7 min. The supernatant was removed and washed with F10/HAM. To minimize fibroblast contamination, the cell suspension was pre-plated in a culture plate for 3 hours in growth media containing 20% FBS, 1% PS and 1% FZ in F10/HAM. The unattached cells were seeded onto Matrigel coated culture flask (0.01% Matrigel in F10/HAM, 30 min at 37°C) and cultured for 4 days in growth media in a humidified incubator with 5% O2 and 5% CO2 at 37°C. After 4 days of incubation, the cell culture medium was changed and then every second day thereafter. At 100% confluence, cells were transferred to intermediate medium (DMEM containing 1 g/L glucose, 10% FBS and 1% PS). After 2 days, medium was changed into differentiation media (DMEM containing 4.5 g/L glucose, 2% horse serum and 1% PS) in order to induce differentiation into myotubes (myocytes). All experiments were performed on fully differentiated myocytes at 7 days of differentiation at passage 4 to 6. For experiments myocytes were serum starved in DMEM containing 1 g/L glucose for 2 hours.

### Glucose Uptake Assay

Cells were treated with or without insulin (100 nM) during the penultimate 30 minutes of serum starvation. Cells were washed twice with Hepes buffered saline (140 mM NaCl, 20 mM HEPES, 5 mM KCl, 2.5 mM MgSO4 and 1 mM CaCl2, pH 7.4). Cells were then incubated with Hepes buffered saline containing 10 µM 2-deoxy-D-[3H] glucose (1 µCi/ml) for 10 min. Nonspecific glucose uptake was determined in the presence of 10 µM cytochalasin B and subtracted from total uptake to get specific glucose uptake. The 2-deoxy-D-[3H] glucose uptake was terminated by removing uptake buffer and washing cells twice with 0.9% ice-cold NaCl. Cells were then solubilized with 50 mM NaOH for 30 min and radioactivity was measured using scintillation counter (Perkin Elmer Tri-Carb 2810TR). Protein concentration was determined using the Bradford reagent. Glucose uptake experiments were carried out in cells from n = 5 individuals per group in triplicate.

### Cell Lysates and Immunoblotting

After treatment, cells were washed in ice-cold phosphate buffered saline (PBS) and lysed with lysis buffer (20 mM Tris, pH 7.5, 150 mM NaCl, 1 mM EGTA, 1 mM EDTA, 0.1% Triton X-100, protease inhibitor (1 tablet/10 ml), 1% phosphatase inhibitor cocktail). Cell lysates were centrifuged at 12000 g, 4°C for 5 min and supernatants were collected. Protein concentration was determined by the Bradford reagent. 20 µg of cell lysates were loaded on 4–15% precast gel (Biorad), transferred to polyvinylidene difluoride (PVDF) membranes and immunoblotted with primary antibodies as indicated in the figure legends. Primary antibodies were detected using appropriate horseradish peroxidase-conjugated secondary IgG antibody. Protein signals visualized using FEMTO enhanced chemiluminescence and Biorad Chemidoc XRS imager. The signal bands were quantified using Image J software (NIH, Bethesda, MD, http://rsb.info.nih.gov/ij). The results were normalized with reactive brown stained total protein.

### RNA Isolation and Real-time PCR

Total RNA was extracted from myocytes using TRIzol according to manufacturer’s instructions. Total RNA was dissolved in RNase-free water and quantified using a Nanodrop ND 1000 (Saveen biotech ApS, Arhus, Denmark). 0.5 µg of total RNA was reverse transcribed using the High Capacity Reverse Transcription kit (Applied Biosystems, Foster City, CA) according to manufacturer’s protocol. Real-time quantitative PCR was performed in triplicate using an ABI-PRISM 7900 (Applied Biosystems). The sequences of the target primers are listed in Table 2. A pre-optimized assay for 18S rRNA was used as endogenous control (Applied Biosystems). Data analysis was performed using the comparative method (ΔΔCT).

**Table 2:** Primer sequences.Immunoflourescence.

mRNA target	Primer(s) 5′-3′ forward	Primer(s) 5′-3′ reverse	probe
hGLUT 1	ACCGGGCCAAGAGTGTGCTA	GTAGGCGGGGGAGCGGAACA	
hGLUT 4	CCTGCCAGAAAGAGTCTGAAGC	ATCCTTCAGCTCAGCCAGCA	CAGAAACATCGGCCCAGCCTGTCA
hMYH1	AGGCAAGCCTGAGGCCACT	CGCTCCCGTTGCCCCAACAA	
hMYH2	TGTCTCACTCCCAGGCTACA	CCAAAAACAGCCAATTCTGAG	

### Immunoflourescence

Myoblasts were differentiated on coverslips pre-coated with poly-L-lysin and Matrigel. At day 7 of differentiation, myotubes were serum starved in DMEM containing 1 g/L glucose for 2 hours before insulin treatment (100 nM insulin, 30 min). Myotubes were fixed with 4% paraformaldehyde in PBS at room temperature for 10 min and then boiled in PBS for 5 min. Subsequently, myotubes were incubated with Alexa Fluor 647-conjugated wheat germ agglutinin for 1 hour, room temperature. Myotubes were then fixed again in 2% paraformaldehyde in PBS for 15 min. Following this membranes were permeabilized and blocked with 4% BSA and 0.2% Triton-x 100 and incubated overnight, 4°C, with anti-GLUT4. Myotubes were subsequently incubated with Alexa Fluor 488-conjugated goat anti-rabbit IgG for 1 hour. After washing, cells were mounted onto slides using Vectashield mounting medium.

### Confocal Microscopy and Image Analysis

Immunofluorescence was analyzed by a Zeiss LSM 710 confocal microscope with 63 X 1.4NA oil-immersion objective. Anti-GLUT4 (Alexa 488) and anti-wheat germ agglutinin (Alexa 647) fluorescence were excited at 488 nm and 633 nm and detected with corresponding channels at 510–530 nm and >660 nm, respectively. Images were recorded at 16 bits and pinhole was set at 2 Airy units. Gain and offset were optimized to obtain best signal from both channels. All images were recorded with identical settings. Quantitative fluorescence intensity was analyzed using image analysis software from NIH. GLUT4 fluorescence signal was measured at the plasma membrane region based on WGA fluorescence signal [25]. Eight to ten cells were analyzed per group (*n* = 5 per groups). Membrane fluorescence intensity (GLUT4 signal at membrane region) was normalized by area and expressed as fold change from basal.

### Statistical Analyses

For multiple comparisons statistical analysis was performed using one-way or two-way analysis of variance (ANOVA) with Bonferroni corrections. For data that was normalized to basal (i.e. fold changes) statistical analysis was performed using a one-sample t-test. Data analysis was performed using GraphPad Prism software and considered statistically significant at P values <0.05.

## Results

### Effect of Aging and Physical Activity on Cell Cycle and Myogenic Markers

**Figure 1:**
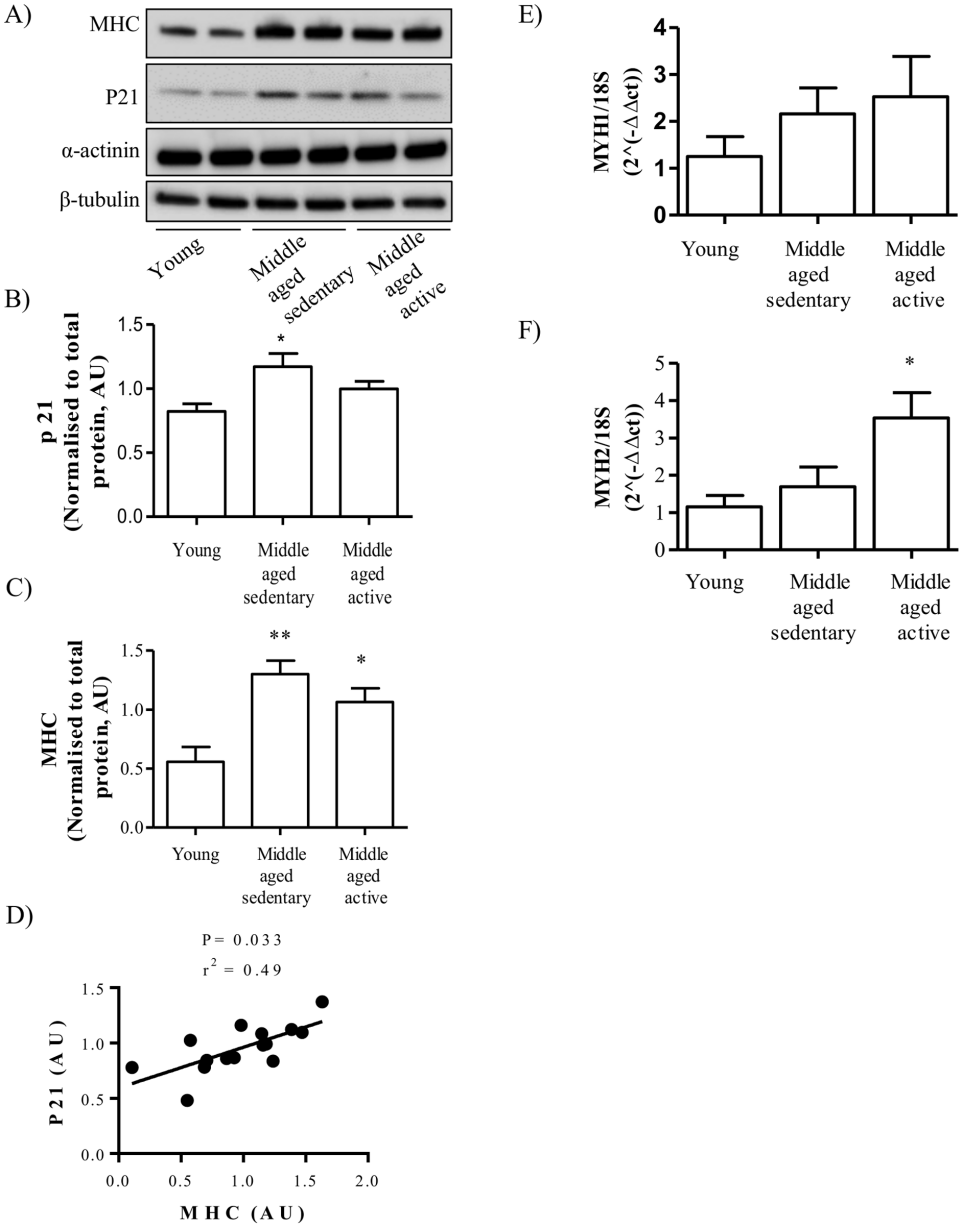
Effect of aging and physical activity on cell cycle and myogenic differentiation. Satellite cells were isolated from vastus lateralis biopsies from young or middle-aged volunteers: sedentary or active. Cells were grown in culture until mature myotubes were formed. (A) Lysates were immunoblotted to assess the total protein amount of MHC, p21 (Waf1/Cip1) and α-actinin. Equal gel loading was ascertained by immunoblotting with an antibody against β-tubulin. Protein expression of (B) p21 and (C) MHC were quantified and expressed as arbitrary units. (D) Protein quantification of MHC was correlated to protein expression of p21 for all groups. Expression of (E) MYH1 and (F) MYH2 were measured by qPCR and using the delta CT method (AU). Values shown are the mean ±S.E.M from cells from 5 individuals for each group. An asterisk denotes a significant difference from young (*P<0.05, **P<0.01).

In order to undergo myogenesis, myoblasts must exit the cell cycle and this is dependent on increased expression of p21 [26], [27]. Conversely, aging is associated with increased expression of p21 in human skeletal muscle [28], [29]. In line with this the protein expression of p21 was significantly increased in myotubes isolated from middle-aged sedentary individuals compared to the expression in myotubes from young individuals (Figure 1A and B). There was no significant difference between p21 protein expression in myotubes from middle-aged active individuals compared to young (P = 0.1). Consistent with increased cell cycle exit, myotubes from middle-aged sedentary and middle-aged active individuals had a significantly higher protein expression of myosin heavy chain (MHC) compared to myotubes from young (Figure 1A and C). There was also a significant increase in p21 protein in middle-aged compared to young irrespective of physical activity level (P = 0.02). Importantly, p21 protein expression correlated to protein expression of MHC in the same cells (Figure 1D). The increase in MHC observed in the middle-aged myotubes represented total sarcomeric myosin heavy chain therefore, in order to look at the composition of myosin heavy chain isoforms in the cells we measured the mRNA level of MYH1, and 2. There was no difference in mRNA expression of MYH1 (type IIb/x fibers) in any group (Figure 1E) however, there was a significant increase in the expression of MYH2 (type IIa fibers) in myotubes from the middle-aged active group compared to the young group (Figure 1F) and a trend towards increased expression compared to the middle-aged sedentary group (P = 0.06).

### Effect of Aging and Physical Activity on Insulin Signaling Components

**Figure 2:**
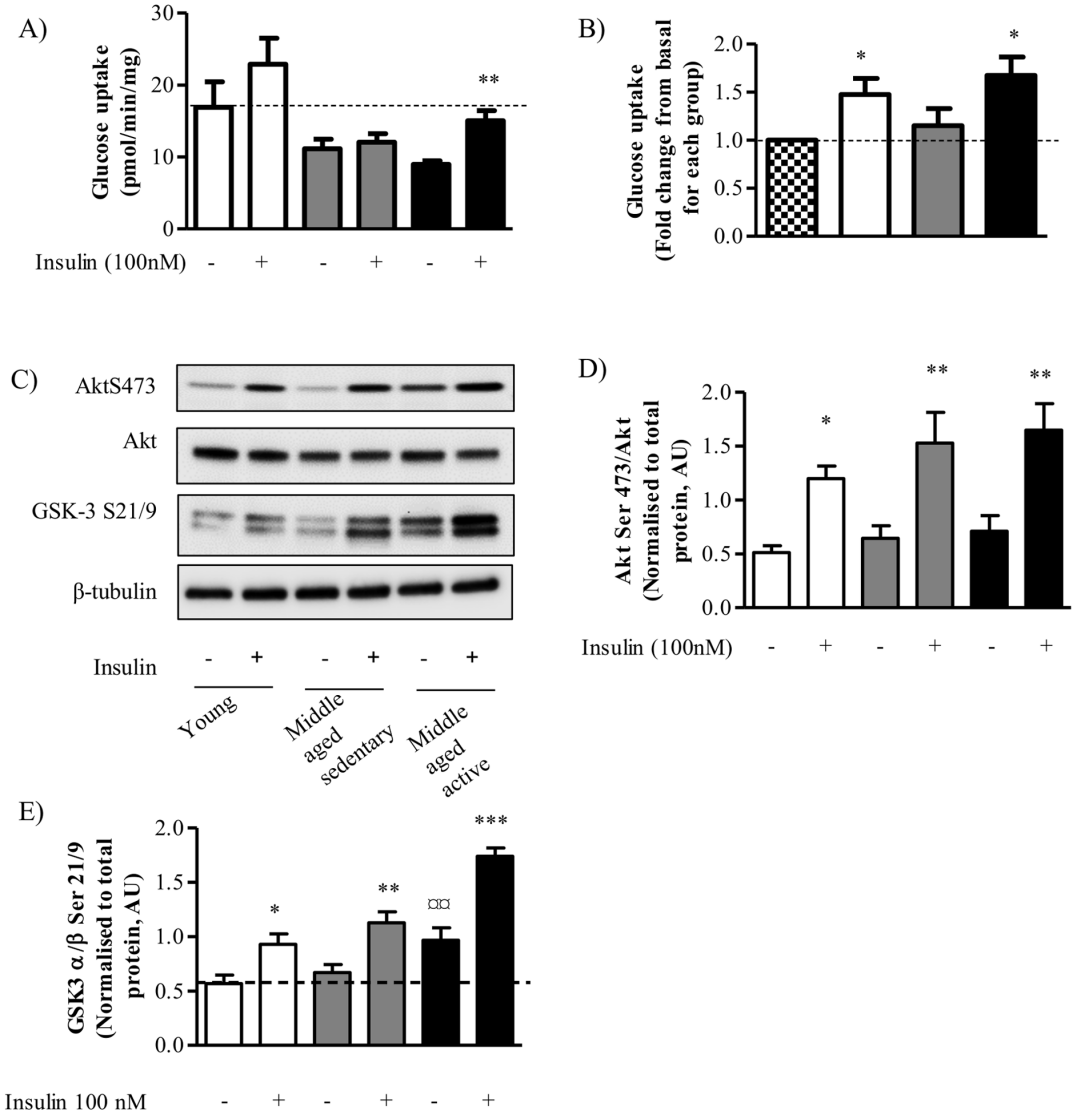
Effect of aging and physical activity on insulin signaling pathway. Satellite cells were isolated from vastus lateralis biopsies from young or middle-aged volunteers: sedentary or active. Cells were grown in culture until mature myotubes were formed. Myotubes were treated with insulin (100 nM) for 30 mins before measuring glucose uptake into myotubes expressed as pmol/min/mg protein (A) or fold change from basal (B). (C) Immunoblotting of total protein lysate for phosphorylation of Akt Serine473 or GSK3 Serine21/9 and total protein amount of Akt. Equal gel loading was ascertained by immunoblotting with an antibody against β-tubulin. Protein expression of (D) Akt phosphorylation and (E) GSK3 phosphorylation were quantified and expressed as arbitrary units. Open bars = young, gray bars = middle-aged sedentary and black bars = middle-aged active. Values shown are the mean ±S.E.M from cells from 5 individuals for each group. An asterisk denotes a significant difference basal for each group (*P<0.05, **P<0.01). A ¤ denotes a significant change from young basal (¤¤ P<0.01).

In order to assess a functional outcome of insulin stimulation in human myocytes we measured the amount of glucose taken up into the cells in the absence and presence of insulin. Myocytes from young and middle-aged active individuals exhibited increased glucose uptake following insulin stimulation (Figure 2A). In myocytes from young individuals we noted a greater inter-subject variation and thus glucose uptake was not significantly induced until adjusting for this variation by instead presenting fold change, which was significantly increased from basal, 1.4 and 1.7 fold increased for young and middle-aged active respectively (Figure 2B). Interestingly, myotubes from middle-aged sedentary individuals did not exhibit any increase in glucose uptake following insulin stimulation (Figure 2A and B), despite having normal glucose plasma values in vivo (Table 1). Interestingly, there was a trend towards a significant difference (P = 0.07) between insulin stimulated glucose uptake level between middle-aged sedentary and middle-aged active myotubes (Figure 2B). In order to understand the mechanism behind this insulin resistant phenotype in the middle-aged sedentary myotubes we measured the phosphorylation of Akt and its downstream target GSK3. In all groups Akt Serine 473 phosphorylation was significantly increased following insulin stimulation (Figure 2C and D). There was no significant difference in the level of Akt protein in any of the groups. In line with intact Akt phosphorylation, insulin-stimulated GSK3 phosphorylation was also significantly increased in all groups (Figure 2C and E). Myotubes from middle-aged active individuals exhibited significantly elevated basal GSK3 phosphorylation compared to young (Figure 2B and E).

### Effect of Aging and Physical Activity on Glucose Transporter Expression

**Figure 3:**
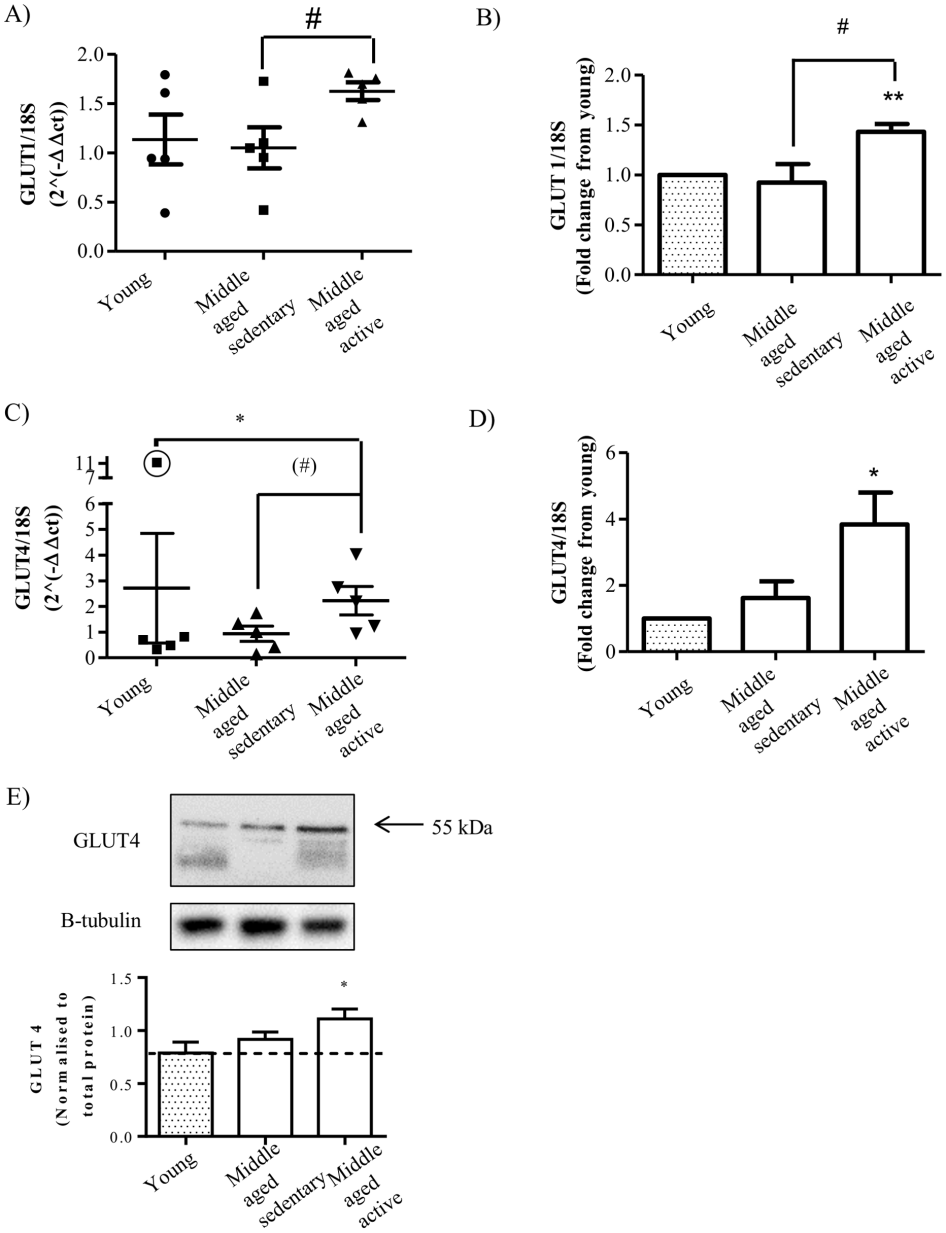
Effect of aging and physical activity on GLUT1 and GLUT4 expression. Satellite cells were isolated from vastus lateralis biopsies from young or middle-aged volunteers: sedentary or active. Cells were grown in culture until mature myotubes were formed. Expression of (A) GLUT1 mRNA and (C) GLUT4 mRNA were measured by qPCR and using the delta CT method (AU). Expression of (B) GLUT1 and (D) GLUT4 mRNA in the middle-aged groups was normalized to expression level in young group. (E) Representative immunoblot and quantification (arbitrary units) of GLUT4 protein. Values shown are the mean ±S.E.M from cells from 5 individuals for each group. An asterisk denotes a significant difference from young (*P<0.05, **P<0.01). A # denotes a significant difference from middle-aged sedentary (P<0.05, t-test). A (#) denotes a trend towards a significant difference from middle-aged sedentary (P = 0.07, t-test). The outlier in GLUT4 is shown with a circle around the value (Figure 3C).

As there were no differences in insulin-stimulated Akt phosphorylation between the groups we measured the expression of glucose transporters (GLUTs). GLUT1, the non-insulin dependent glucose transporter, was significantly increased at the mRNA level in middle-aged active myotubes compared to both middle-aged sedentary myotubes (Figure 3A) and young myotubes (Figure 3B). GLUT4, the insulin sensitive glucose transporter, showed a trend towards increased mRNA expression in middle-aged active myotubes compared to middle-aged sedentary (Figure 3C). GLUT4 expression in young myotubes had one subject who expressed 11 and 6 fold higher mRNA expression than the rest in two experiments respectively and was identified as an outlier using graph pad prism 6 software, therefore this individual was excluded from the statistical analysis and fold change analysis (Figure 3C and D). GLUT4 expression in middle-aged active myotubes was significantly higher than in young when the outlier was excluded (Figure 3 C and D) and there was a trend (P = 0.073) towards increased expression in middle-aged active compared to middle-aged sedentary myotubes. In line with the increased mRNA expression, myotubes from middle-aged active individuals had significantly higher GLUT4 protein expression compared to the young group (Figure 3E) and there was no difference between young and middle-aged sedentary.

### Effect of Aging and Physical Activity on Insulin-stimulated GLUT4 Localization

**Figure 4:**
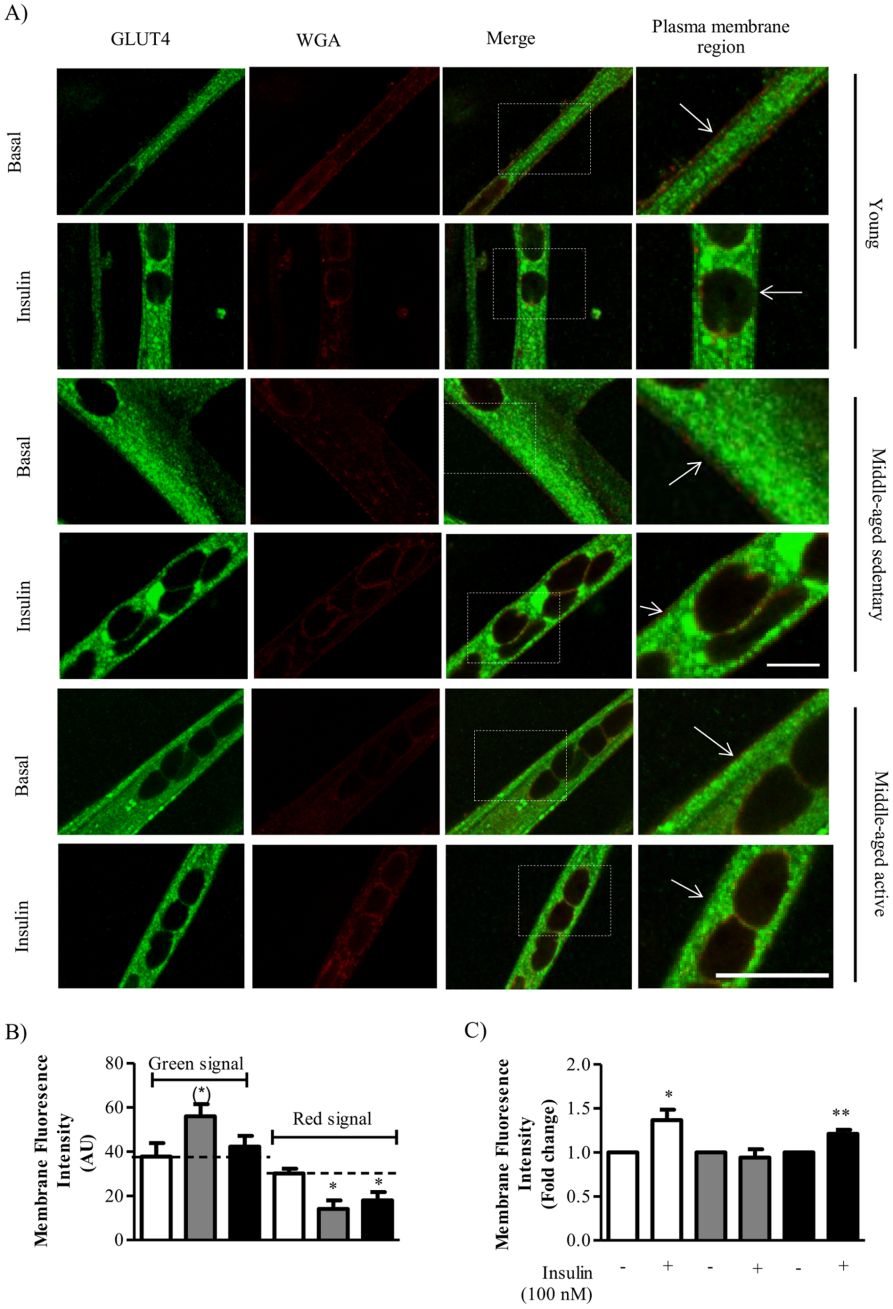
Effect of aging and physical activity on insulin-stimulated GLUT4 localisation. Satellite cells were isolated from vastus lateralis biopsies from young or middle-aged volunteers: sedentary or active. Cells were grown in culture until mature myotubes were formed. Myotubes were treated with insulin (100 nM) for 30 mins before fixation and immunofluorescence staining. (A) Representative immunofluorescence images of myotubes stained with anti-GLUT4 and anti-wheat germ agglutinin (Scale bar 20 µm). The plasma membrane region is a 2× zoom of the merged image. Quantification of membrane fluorescence intensity in basal state (B) or after insulin treatment as a fold change from basal (C). (B and C) Open bars = young, gray bars = middle-aged sedentary and black bars = middle-aged active. Values are mean ± S.E.M from cells from 5 individuals for each group. An asterisk denotes a significant difference from basal (*P<0.05, **P<0.01). A (*) denotes a trend towards a significant difference from young (P = 0.058).

In order to further understand the mechanism by which physical activity protects against aging associated insulin resistance we investigated GLUT4 translocation in myotubes, in the absence or presence of insulin, by immunofluorescence (Figure 4A). In order to quantify the amount of GLUT4 at the plasma membrane we also stained with wheat germ agglutinin before permeabilization of the plasma membrane (Figure 4A). Myotubes from both middle-aged sedentary and middle-aged active individuals had significantly lower levels of the (plasma membrane marker) WGA staining compared to myotubes from young individuals (Figure 4B). Myotubes from middle-aged sedentary individuals showed a borderline significant (P = 0.058) increase in GLUT4 at the plasma membrane in the basal state compared to young myotubes (Figure 4B). GLUT4 signal at the plasma membrane in the presence of insulin was normalized to signal at plasma membrane at basal for each group and showed that myotubes from young subjects and middle-aged active subjects had a significant increase in GLUT4 signal following insulin stimulation (Figure 4C). Interestingly, myotubes from middle-aged sedentary subjects showed no detectable increase in GLUT4 signal at the plasma membrane following insulin treatment (Figure 4C).

## Discussion

Our findings indicate that human skeletal muscle satellite cells that are grown and differentiated into myotubes in culture, despite multiple passages, exhibit metabolic differences associated with level of activity and age of donors. This result suggests that differentiated myotubes may retain memory of their in vivo environment. Interestingly, a number of metabolic factors were significantly enhanced in middle-aged active cells compared to young healthy controls suggesting that life-long physical activity promotes adaptations that robustly alter metabolism of muscle cells and is retained *ex vivo*. Therefore our model may help decipher the mechanisms by which physical activity potentially can protect against the development of insulin resistance.

In order to undergo myogenesis, myoblasts must exit the cell cycle and this is dependent on increased expression of p21 [26], [27]; the expression of p21 is therefore indicative of irreversible cell cycle exit required for terminal differentiation. Consistent with this, increased expression of cyclin dependent kinase (CDK) inhibitor p21 (WAF1/CIP1) has been found in skeletal muscle of aging mice [30] and in human skeletal muscle during aging (22, 23). Both p21 and MHC have been shown to be highly upregulated in muscles of older women (65–71 years) [29]. Additionally p21 is known to be upregulated during myocyte differentiation in myogenic cell lines [31], [32]. Importantly it has been shown that MHC expression is only detected in p21-positive cells [33]. In line with this we found that middle-aged cells expressed higher protein levels of p21 and MHC compared to the young group and their expressions were strongly correlated. Further analysis of MHC isoforms showed different expression of MHC isoforms in sedentary and active middle-aged muscle cells. We found that myotubes from middle-aged active individuals expressed significantly higher mRNA levels of MYH2 compared to myotubes from young individuals suggesting a training or adaptation effect of physical activity. MYH2 is a marker of type IIa muscle fibers (fast, oxidative fibers). Interestingly, it has been shown that in rats, type IIa fibers have a higher glucose uptake capacity than type IIb/x fibers (MYH1) [34] therefore this could contribute to the preserved insulin sensitivity in middle-aged active myotubes in this study. Increased MHCIIa has been shown to occur in both men and women who have undergone resistance exercise training [35], [36] and importantly has been shown to increase in old men after aerobic exercise training [37]. It has been reported that in obese and type 2 diabetics there is an inverse relationship between insulin sensitivity and the percent of myosin heavy chain IIx (MYH1) [38]. However, in this study we did not observe any difference in MYH1 expression in any of the groups.

GLUT4 is expressed exclusively in skeletal muscle and fat cells as they are the main sites for glucose disposal [39]. It has been well documented that both endurance and resistance exercise induce increases in GLUT4 protein expression allowing for increased glucose uptake and glycogen storage in muscle [40], [41], [42], [43]. However, our findings of significant increases in GLUT4 protein and mRNA in the middle-aged active group compared to both young and middle-aged sedentary groups in isolated satellite cells that have been expanded and differentiated in vitro, suggesting in part that the cells retain differences associated with physical activity. Interestingly, Stuart et al have shown that 6 weeks of progressive cycle training in sedentary individuals primarily increases GLUT4 expression in Type II fibers [44]. This finding is in line with our results that both MYH2 and GLUT4 expression levels are increased in myotubes from middle-aged active individuals. As a consequence of increased GLUT4 expression following glycogen depleting exercise, glycogen storage occurs more rapidly and to a greater extent in the trained state versus the untrained state [45]. In this study we found that in myotubes from middle-aged active individuals there was a significantly elevated level of GSK3 phosphorylation in the basal state compared to the level in the young group again suggesting an adaptation to life-long training that enhances muscle metabolism above that of a younger, untrained individual. As GSK3 phosphorylation results in increased glycogen synthase activity and subsequent glycogen storage this finding may reflect the exercise mediated training adaptation that has been observed *in vivo*.

As the protein expression of GLUT4 in the middle-aged sedentary myotubes was also comparable to young we investigated the effects of aging and physical activity on insulin-stimulated GLUT4 translocation to the plasma membrane. Interestingly, in the basal state we found increased GLUT4 fluorescence signal at the plasma membrane in the middle-aged sedentary myotubes which may reflect a dysregulated trafficking or recycling of GLUT4 in these cells. GLUT4 cycling between cellular compartments and the plasma membrane is a complex, multi-step process that involves translocation, docking and fusion with the plasma membrane, endocytosis and recycling through the endoplasmic reticulum therefore the potential defect in middle-aged sedentary cells could occur at multiple levels of this cycle. Interestingly, both middle-aged sedentary and middle-aged active myotubes had observable (and significant) decreases in WGA signal. As WGA selectively binds to sialic acid and N-acetylglucosaminyl residues that are predominantly found in the plasma membrane [46] it is widely used as a marker of the plasma membrane in microscopy. Thus, the decrease in WGA signal in the middle-aged myotubes could reflect a decrease in integrity or dysregulation of the plasma membrane however, further investigation is required to confirm this. Importantly, following insulin stimulation, myotubes from both young and middle-aged active individuals had significantly increased GLUT4 signal at the plasma membrane compared to basal; indicating intact insulin-stimulated GLUT4 translocation. However, in myotubes from middle-aged sedentary cells there was no increase in GLUT4 signal at the plasma membrane following insulin treatment. As the basal level at the plasma membrane was high in this group this may reflect impaired docking or fusion of GLUT4 at the plasma membrane and that physical activity protects against or promotes adaptations that protect against impaired GLUT4 cycling during age.

GLUT1 is a non-insulin dependent glucose transporter that is ubiquitously expressed and has a low expression in skeletal muscle [47] however, in this study we observed a significant increase in its mRNA level in myotubes from middle-aged active individuals. It has been suggested that GLUT1 plays an important role in basal (non-insulin stimulated) glucose uptake in skeletal muscle [48] however, increased mRNA expression of GLUT1 in middle-aged active myotubes in this study, was not accompanied by an increase in basal glucose uptake but rather a trend towards decreased basal glucose uptake. There also seemed to be a lower basal glucose uptake in middle-aged sedentary myotubes compared to young myotubes and this may reflect the potential decrease in membrane integrity in both middle-aged groups.

### Conclusion

In conclusion, human myotubes in culture obtained from middle-aged sedentary individuals have differences in insulin stimulated glucose metabolism that would be expected to be seen due to secondary aging in vivo, including impaired insulin-stimulated glucose uptake. Interestingly, physical activity throughout life seems to protect myotubes from these aspects of secondary aging potentially through adaptations including increased expression of GLUT4 and MYH2. Additionally, lifelong physical activity exerts positive effects on muscle metabolism (including enhanced GSK3 phosphorylation and GLUT4 expression) when compared to the same parameters in myotubes from young, recreationally active, healthy controls. We also found that impaired insulin-stimulated glucose uptake in middle-aged sedentary myotubes was unlikely to be due to decreased GLUT4 protein expression and is likely to be due to dysregulated GLUT4 cycling to the plasma membrane.

## References

[1] Meigs JB, Muller DC, Nathan DM, Blake DR, Andres R (2003) The natural history of progression from normal glucose tolerance to type 2 diabetes in the Baltimore Longitudinal Study of Aging. Diabetes 52: 1475–1484. 12765960 10.2337/diabetes.52.6.1475

[2] Nichols GA, Hillier TA, Brown JB (2007) Progression from newly acquired impaired fasting glusose to type 2 diabetes. Diabetes Care 30: 228–233. 17259486 10.2337/dc06-1392 PMC1851903

[3] Weyer C, Bogardus C, Mott DM, Pratley RE (1999) The natural history of insulin secretory dysfunction and insulin resistance in the pathogenesis of type 2 diabetes mellitus. J.Clin.Invest 104: 787–794. 10491414 10.1172/JCI7231 PMC408438

[4] Thyfault JP, Booth FW (2011) Lack of regular physical exercise or too much inactivity. Curr.Opin.Clin.Nutr.Metab Care 14: 374–378. 21519238 10.1097/MCO.0b013e3283468e69

[5] Carmeli E, Reznick AZ (1994) The physiology and biochemistry of skeletal muscle atrophy as a function of age. Proc.Soc.Exp.Biol.Med. 206: 103–113. 10.3181/00379727-206-43727 8208732

[6] Tuomilehto J, Lindstrom J, Eriksson JG, Valle TT, Hamalainen H, et al (2001) Prevention of type 2 diabetes mellitus by changes in lifestyle among subjects with impaired glucose tolerance. N.Engl.J.Med. 344: 1343–1350. 10.1056/NEJM200105033441801 11333990

[7] Booth FW, Laye MJ, Roberts MD (2011) Lifetime sedentary living accelerates some aspects of secondary aging. J.Appl.Physiol 111: 1497–1504. 21836048 10.1152/japplphysiol.00420.2011

[8] Holloszy JO (2000) The biology of aging. Mayo Clin.Proc. 75 Suppl: S3–S8 10959208

[9] Fink RI, Kolterman OG, Griffin J, Olefsky JM (1983) Mechanisms of insulin resistance in aging. J.Clin.Invest 71: 1523–1535. 6345584 10.1172/JCI110908 PMC370358

[10] Carrascosa JM, Andres A, Ros M, Bogonez E, Arribas C, et al (2011) Development of insulin resistance during aging: involvement of central processes and role of adipokines. Curr.Protein Pept.Sci. 12: 305–315. 10.2174/138920311795906655 21574953

[11] Seals DR, Hagberg JM, Allen WK, Hurley BF, Dalsky GP, et al (1984) Glucose tolerance in young and older athletes and sedentary men. J.Appl.Physiol 56: 1521–1525. 6376436 10.1152/jappl.1984.56.6.1521

[12] Balagopal P, Schimke JC, Ades P, Adey D, Nair KS (2001) Age effect on transcript levels and synthesis rate of muscle MHC and response to resistance exercise. Am.J.Physiol Endocrinol.Metab 280: E203–E208. 11158921 10.1152/ajpendo.2001.280.2.E203

[13] Menshikova EV, Ritov VB, Fairfull L, Ferrell RE, Kelley DE, et al (2006) Effects of exercise on mitochondrial content and function in aging human skeletal muscle. J.Gerontol.A Biol.Sci.Med.Sci. 61: 534–540. 10.1093/gerona/61.6.534 PMC1540458 16799133

[14] Kahn SE, Larson VG, Beard JC, Cain KC, Fellingham GW, et al (1990) Effect of exercise on insulin action, glucose tolerance, and insulin secretion in aging. Am.J.Physiol 258: E937–E943. 2193534 10.1152/ajpendo.1990.258.6.E937

[15] MAURO A (1961) Satellite cell of skeletal muscle fibers. J.Biophys.Biochem.Cytol. 9: 493–495. 10.1083/jcb.9.2.493 PMC2225012 13768451

[16] Robson LG, Di F, V, Radunovic A, Bird K, Zhang X et al. (2011) Bmi1 is expressed in postnatal myogenic satellite cells, controls their maintenance and plays an essential role in repeated muscle regeneration. PLoS.One. 6: e27116–. 10.1371/journal.pone.0027116 PMC3212532 22096526

[17] Alfaro LA, Dick SA, Siegel AL, Anonuevo AS, McNagny KM, et al (2011) CD34 promotes satellite cell motility and entry into proliferation to facilitate efficient skeletal muscle regeneration. Stem Cells 29: 2030–2041. 21997891 10.1002/stem.759 PMC3638793

[18] Sambasivan R, Yao R, Kissenpfennig A, Van WL, Paldi A, et al (2011) Pax7-expressing satellite cells are indispensable for adult skeletal muscle regeneration. Development 138: 3647–3656. 21828093 10.1242/dev.067587

[19] Gaster M, Kristensen SR, Beck-Nielsen H, Schroder HD (2001) A cellular model system of differentiated human myotubes. APMIS 109: 735–744. 11900052 10.1034/j.1600-0463.2001.d01-140.x

[20] Green CJ, Pedersen M, Pedersen BK, Scheele C (2011) Elevated NF-kappaB activation is conserved in human myocytes cultured from obese type 2 diabetic patients and attenuated by AMP-activated protein kinase. Diabetes 60: 2810–2819. 21911750 10.2337/db11-0263 PMC3198079

[21] Gaster M, Petersen I, Hojlund K, Poulsen P, Beck-Nielsen H (2002) The diabetic phenotype is conserved in myotubes established from diabetic subjects: evidence for primary defects in glucose transport and glycogen synthase activity. Diabetes 51: 921–927. 11916908 10.2337/diabetes.51.4.921

[22] Bouzakri K, Roques M, Gual P, Espinosa S, Guebre-Egziabher F, et al (2003) Reduced activation of phosphatidylinositol-3 kinase and increased serine 636 phosphorylation of insulin receptor substrate-1 in primary culture of skeletal muscle cells from patients with type 2 diabetes. Diabetes 52: 1319–1325. 12765939 10.2337/diabetes.52.6.1319

[23] Scheele C, Nielsen S, Kelly M, Broholm C, Nielsen AR et al. (2012) Satellite cells derived from obese humans with type 2 diabetes and differentiated into myocytes in vitro exhibit abnormal response to IL-6. PLoS.One. 7: e39657–. 10.1371/journal.pone.0039657 PMC3383673 22761857

[24] Broholm C, Brandt C, Schultz NS, Nielsen AR, Pedersen BK, et al (2012) Deficient leukemia inhibitory factor signaling in muscle precursor cells from patients with type 2 diabetes. Am.J.Physiol Endocrinol.Metab 303: E283–E292. 22649064 10.1152/ajpendo.00586.2011

[25] Graveleau C, Zaha VG, Mohajer A, Banerjee RR, Dudley-Rucker N, et al (2005) Mouse and human resistins impair glucose transport in primary mouse cardiomyocytes, and oligomerization is required for this biological action. J.Biol.Chem. 280: 31679–31685. 10.1074/jbc.M504008200 15983036

[26] Guo K, Wang J, Andres V, Smith RC, Walsh K (1995) MyoD-induced expression of p21 inhibits cyclin-dependent kinase activity upon myocyte terminal differentiation. Mol.Cell Biol. 15: 3823–3829. 10.1128/mcb.15.7.3823 PMC230621 7791789

[27] Halevy O, Novitch BG, Spicer DB, Skapek SX, Rhee J, et al (1995) Correlation of terminal cell cycle arrest of skeletal muscle with induction of p21 by MyoD. Science 267: 1018–1021. 7863327 10.1126/science.7863327

[28] Welle S, Brooks AI, Delehanty JM, Needler N, Thornton CA (2003) Gene expression profile of aging in human muscle. Physiol Genomics 14: 149–159. 12783983 10.1152/physiolgenomics.00049.2003

[29] Welle S, Brooks AI, Delehanty JM, Needler N, Bhatt K, et al (2004) Skeletal muscle gene expression profiles in 20–29 year old and 65–71 year old women. Exp.Gerontol. 39: 369–377. 10.1016/j.exger.2003.11.011 15036396

[30] Edwards MG, Anderson RM, Yuan M, Kendziorski CM, Weindruch R et al. (2007) Gene expression profiling of aging reveals activation of a p53-mediated transcriptional program. BMC.Genomics 8: 80–. 10.1186/1471-2164-8-80 PMC1847444 17381838

[31] Guo K, Wang J, Andres V, Smith RC, Walsh K (1995) MyoD-induced expression of p21 inhibits cyclin-dependent kinase activity upon myocyte terminal differentiation. Mol.Cell Biol. 15: 3823–3829. 10.1128/mcb.15.7.3823 PMC230621 7791789

[32] Halevy O, Novitch BG, Spicer DB, Skapek SX, Rhee J, et al (1995) Correlation of terminal cell cycle arrest of skeletal muscle with induction of p21 by MyoD. Science 267: 1018–1021. 7863327 10.1126/science.7863327

[33] Andres V, Walsh K (1996) Myogenin expression, cell cycle withdrawal, and phenotypic differentiation are temporally separable events that precede cell fusion upon myogenesis. J.Cell Biol. 132: 657–666. 10.1083/jcb.132.4.657 PMC2199863 8647896

[34] Mackrell JG, Arias EB, Cartee GD (2012) Fiber type-specific differences in glucose uptake by single fibers from skeletal muscles of 9- and 25-month-old rats. J.Gerontol.A Biol.Sci.Med.Sci. 67: 1286–1294. 10.1093/gerona/gls194 PMC3670157 23042591

[35] Bamman MM, Hill VJ, Adams GR, Haddad F, Wetzstein CJ, et al (2003) Gender differences in resistance-training-induced myofiber hypertrophy among older adults. J.Gerontol.A Biol.Sci.Med.Sci. 58: 108–116. 10.1093/gerona/58.2.b108 12586847

[36] Liu Y, Heinichen M, Wirth K, Schmidtbleicher D, Steinacker JM (2008) Response of growth and myogenic factors in human skeletal muscle to strength training. Br.J.Sports Med. 42: 989–993. 10.1136/bjsm.2007.045518 18308879

[37] Harber MP, Konopka AR, Undem MK, Hinkley JM, Minchev K, et al (2012) Aerobic exercise training induces skeletal muscle hypertrophy and age-dependent adaptations in myofiber function in young and older men. J.Appl.Physiol 113: 1495–1504. 22984247 10.1152/japplphysiol.00786.2012 PMC3524668

[38] Gjovaag TF, Dahl HA (2009) Effect of training with different mechanical loadings on MyHC and GLUT4 changes. Med.Sci.Sports Exerc. 41: 129–136. 10.1249/MSS.0b013e3181844e42 19092697

[39] Guillet-Deniau I, Leturque A, Girard J (1994) Expression and cellular localization of glucose transporters (GLUT1, GLUT3, GLUT4) during differentiation of myogenic cells isolated from rat foetuses. J.Cell Sci. 107 (Pt 3): 487–496. 8006068

[40] Dela F, Ploug T, Handberg A, Petersen LN, Larsen JJ, et al (1994) Physical training increases muscle GLUT4 protein and mRNA in patients with NIDDM. Diabetes 43: 862–865. 8013748 10.2337/diab.43.7.862

[41] Goodyear LJ, Hirshman MF, Valyou PM, Horton ES (1992) Glucose transporter number, function, and subcellular distribution in rat skeletal muscle after exercise training. Diabetes 41: 1091–1099. 1323491 10.2337/diab.41.9.1091

[42] Rodnick KJ, Holloszy JO, Mondon CE, James DE (1990) Effects of exercise training on insulin-regulatable glucose-transporter protein levels in rat skeletal muscle. Diabetes 39: 1425–1429. 2227115 10.2337/diab.39.11.1425

[43] Christ-Roberts CY, Pratipanawatr T, Pratipanawatr W, Berria R, Belfort R, et al (2004) Exercise training increases glycogen synthase activity and GLUT4 expression but not insulin signaling in overweight nondiabetic and type 2 diabetic subjects. Metabolism 53: 1233–1242. 15334390 10.1016/j.metabol.2004.03.022

[44] Stuart CA, Howell ME, Baker JD, Dykes RJ, Duffourc MM, et al (2010) Cycle training increased GLUT4 and activation of mammalian target of rapamycin in fast twitch muscle fibers. Med.Sci.Sports Exerc. 42: 96–106. 10.1249/MSS.0b013e3181ad7f36 PMC2796589 20010125

[45] Nakatani A, Han DH, Hansen PA, Nolte LA, Host HH, et al (1997) Effect of endurance exercise training on muscle glycogen supercompensation in rats. J.Appl.Physiol 82: 711–715. 9049757 10.1152/jappl.1997.82.2.711

[46] Nagata Y, Burger MM (1974) Wheat germ agglutinin. Molecular characteristics and specificity for sugar binding. J.Biol.Chem. 249: 3116–3122. 4830237

[47] Gaster M, Franch J, Staehr P, Beck-Nielsen H, Smith T, et al (2000) Induction of GLUT-1 protein in adult human skeletal muscle fibers. Am.J.Physiol Endocrinol.Metab 279: E1191–E1195. 11052976 10.1152/ajpendo.2000.279.5.E1191

[48] Ciaraldi TP, Mudaliar S, Barzin A, Macievic JA, Edelman SV, et al (2005) Skeletal muscle GLUT1 transporter protein expression and basal leg glucose uptake are reduced in type 2 diabetes. J.Clin.Endocrinol.Metab 90: 352–358. 15483099 10.1210/jc.2004-0516

